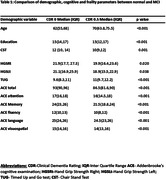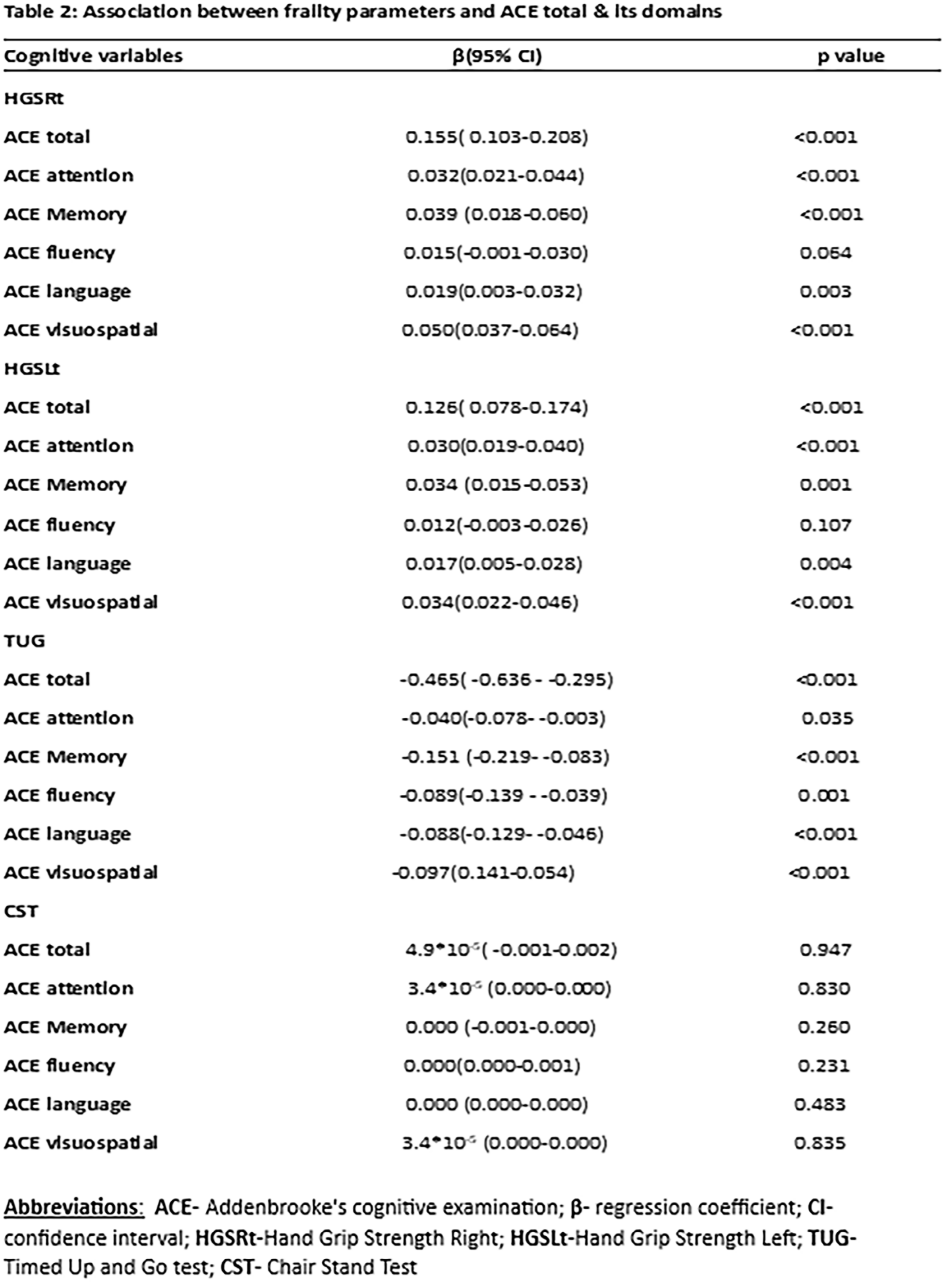# Frailty and Cognition: Unravelling the Intricate Nexus

**DOI:** 10.1002/alz.091190

**Published:** 2025-01-03

**Authors:** Ajith Partha, Monisha S, Albert Stezin, Abhishek M L, Divya N Mallikarjun, Amitha C M, Deva Kumar HS, Rajitha Narayanasamy, Meenakshi Menon, Meghana R, Vindhya Vishwanath, Goutham Velavarajan, Palash K Malo, Prathima Arvind, Shafeeq K Shahul Hameed, Sunitha HS, Sadhana Singh, Banashree Mondal, Deepashri Agrawal, Jonas S. Sundarakumar, Thomas Gregor Issac

**Affiliations:** ^1^ Centre for Brain Research, Indian Institute of Science, Bangalore, Karnataka India

## Abstract

**Background:**

Frailty, synonymous with physical vulnerability and decline, may exert nuanced effects on cognitive functions.(Borges, Canevelli, Cesari, & Aprahamian, 2019) This study assesses possible association between frailty and cognitive performance with the aim of identifying vulnerable cognitive domains.

**Method:**

We analysed the baseline data from Tata Longitudinal Study of Ageing (TLSA)(Sundarakumar et al., 2020), an ongoing longitudinal study, based in urban Bangalore, India. Frailty was evaluated using clinical markers such as Timed Up and Go test (TUG), Hand Grip Strength right and left (HGSRt and HGSLt respectively), and Chair Stand test (CST). Cognitive function was evaluated using Addenbrooke’s Cognitive Examination III (ACE III), and Clinical Dementia Rating (CDR) scores. Generalized linear models/ Binary logistic regression were used to evaluate the relationship between frailty parameters and cognition.

**Result:**

In a cohort of 1484 participants (51% males, 49% females; mean age 62.3 ± 9.6, mean education 15.2±3.8 years), significant positive correlation was found between HGS and ACE scores and significant negative correlation was found between TUG and ACE scores. Notably, no significant association was found between the Chair Stand Test (CST) and ACE scores (Table 1&2). Individuals with higher HGSRt (Odds Ratio [OR] = 0.967, 0.939‐0.996, p value 0.026), and CST (OR = 0.903, 0.839‐0.972, p value 0.006) exhibited a lower likelihood of Mild Cognitive Impairment (MCI). Additionally, those with MCI took longer duration in TUG test (OR = 1.209, 1.122‐1.304, p value <0.001) compared to normal participants. There was no significant association between MCI and HGSLt (OR = 0.973, 0.945‐1.002, p value <0.069).

**Conclusion:**

The study reveals a significant association between frailty parameters and cognitive functions. These findings demonstrate the impact of physical resilience on cognitive well‐being, emphasizing the importance of addressing frailty as a modifiable factor in promoting cognitive health among aging individuals.